# Correlates of health-related quality of life among adults receiving combination antiretroviral therapy in coastal Kenya

**DOI:** 10.1186/s12955-020-01421-0

**Published:** 2020-06-05

**Authors:** Moses K. Nyongesa, Paul Mwangi, Stanley W. Wanjala, Agnes M. Mutua, Hans M. Koot, Pim Cuijpers, Charles R. J. C. Newton, Amina Abubakar

**Affiliations:** 1 Neurosciences Group, KEMRI/Wellcome Trust Research Programme, Centre for Geographic Medicine Research (Coast), Kilifi, Kenya; 2 Department of Clinical, Neuro- and Developmental Psychology, Amsterdam Public Health Research Institute, Vrije Universiteit Amsterdam, Amsterdam, Netherlands; 3 Department of Social Sciences, Pwani University, Kilifi, Kenya; 4 Department of Public Health, Pwani University, Kilifi, Kenya; 5 Department of Psychiatry, University of Oxford, Oxford, UK; 6 Institute for Human Development, Aga Khan University, Nairobi, Kenya

**Keywords:** HIV infections, Adults, Quality of life, Correlates, Psychometrics, Antiretroviral therapy, Kenya

## Abstract

**Background:**

Health-related quality of life (HRQoL) is an important metric of perceived wellbeing in people living with HIV/AIDS (PLWHA). However, research on HRQoL among PLWHA in sub-Saharan Africa is limited. This study investigates factors associated with HRQoL among PLWHA in Kilifi, coast of Kenya.

**Methods:**

Between February and April 2018, 450 adults living with HIV and on combined antiretroviral therapy (cART) between 18 to 60 years were sequentially recruited from an HIV-specialized clinic. The Functional Assessment of HIV Infection (FAHI) questionnaire, previously adapted for assessing HRQoL in this setting, was slightly modified and administered to participants alongside other measures of sociodemographic, health and treatment characteristics in a face-to-face interview.

**Results:**

Linear regression analyses indicated that depressive symptoms, HIV-related stigma, non-disclosure of HIV status, living alone, clinic inaccessibility, and presence of any current opportunistic infection were significantly associated with lower HRQoL scores at both the FAHI overall and sub-scale level. Higher physician empathy, male sex, and higher body mass index were significantly associated with better HRQoL scores at both FAHI overall and sub-scale level. Age and longer duration on cART were significantly associated with better HRQoL only at the sub-scale level.

**Conclusions:**

Interventions aimed at reducing depressive symptoms and HIV stigma, making HIV-related services more accessible, addressing opportunistic infections, strengthening social support systems, serostatus disclosure and put in place caring, respectful, and compassionate model of care are necessary to improve the HRQoL of PLWHA.

## Introduction

As of 2017, 36.9 million people were living with HIV, with nearly 2 million new HIV infections in the same year [1]. Sub-Saharan Africa (SSA) continues to bear the greatest HIV-related burden as over half of people living with HIV/AIDS (PLWHA) worldwide reside in this region [1]. Nevertheless, strides have been made globally to increase the availability and accessibility of combined antiretroviral therapy (cART) for the management of HIV/AIDS even in low-resource settings of SSA [1]. In Kenya, cART coverage is reported as 75% among adults [2].

Owing to cART and its adherence, HIV-related morbidity and mortality have declined enormously [3, 4] and life expectancy of PLWHA has improved [5, 6]. Moreover, with the advent of cART, HIV – previously considered a fatal disease – is now managed like any other incurable chronic condition [7, 8]. Following the achieved milestones and the evolution of HIV to a chronic and potentially manageable disease, research interest is shifting to the understanding of the impact of this illness and its treatment on long-term outcomes such as Health-related Quality of Life (HRQoL).

HRQoL is multi-dimensional and can be understood as the patients’ subjective evaluation of themselves based on perception of the impact of illness and/or its treatment on their wellbeing [9, 10] in the physical, emotional, social, cognitive, functional and global domains. It is an important clinical metric of perceived wellbeing in PLWHA [11]. Evaluating HRQoL of PLWHA in research and clinical practice may provide important complementary information on patient wellbeing that is not captured by the often-used biological markers of immune status, namely cluster of differentiation-4 (CD4) cell count and serum viral load. These biological markers evaluate HIV progression, but rarely capture the patient’s experience of treatment benefit [12]. For PLWHA, attaining longer life expectancy with antiretroviral treatment is more salient if they can sustain optimum levels of HRQoL [13].

Several measures are available for assessing HRQoL of PLWHA. These include HIV-specific instruments such as the medical outcomes study-HIV [14], the HIV overview of problems evaluation system [15], the World Health Organization Quality of Life instrument module for international assessment of HIV/AIDS [16], and the Functional Assessment of HIV Infection [17]. There is no “gold standard” HRQoL instrument for use with PLWHA per se. The choice really depends on the purpose of the quality of life assessment and the most relevant domains of HRQoL that are specific to research or clinical questions. Noteworthy is that most of these existing HRQoL measures have been developed, validated and used in high-resource settings. However, validation work emerging from low-resource settings (e.g., Nyongesa et al. [18]), demonstrates that it is possible to contextually adapt and use these measures.

Various studies, mostly from high-resource settings (see review by Degroote et al. [8]), but also emerging reports from SSA [19–22], have investigated factors associated with HRQoL among PLWHA. From these studies, factors related to HRQoL in PLWHA can broadly be categorized as: sociodemographic (such as, age, sex, socioeconomic status, and family situation; although mixed evidence); clinical and disease-related (such as, viral load, CD4 cell count, antiretroviral therapy, disclosure, and disease stage and symptoms); psychological (such as, depressive symptoms, coping strategy, social support, and HIV-related stigma); and behavioural (such as, smoking, alcohol and other substance abuse, and cART adherence). Most of these studies report a negative association between HRQOL and having depressive symptoms or experiencing HIV-related stigma; whereas mixed finding is reported for the other variables.

Although HRQoL is an important metric in the course of HIV disease [11], and should be a primary area of focus when providing care and support to PLWHA [23]; overall, little research has been conducted to look at HRQoL and its associated factors among PLWHA in the SSA context. This study aimed at investigating sociodemographic, clinical and disease-related, psychological, and behavioural factors as candidate correlates of HRQoL of adults living with HIV/AIDs from Coastal Kenya who are under cART management. To our knowledge, the present study is the first to investigate the HRQoL of PLHIV in the Coastal region of Kenya using an HIV-specific assessment measure. In this setting, with a population of 1.4 million people, the prevalence of adult HIV is estimated at 5% [24]. This study, in addition to emerging reports within SSA [19–22] adds to the accumulating body of knowledge about HRQoL of PLWHA living in resource-limited settings. Studies on correlates of HRQoL are key to informing better clinical care practices, identifying those in need of extra support or context-specific priority areas for intervention. The latter is particularly important in settings where the disease burden is high, yet resources are limited.

## Methods

### Study setting and participants

This work was part of data collected from a larger cross-sectional study looking at different outcomes in adults living with HIV such as mental health, HIV stigma and HRQoL. The study was conducted at the Centre for Geographic Medicine Research (CGMR), located in Kilifi County, Coastal Kenya between 20th February and 15th April 2018. Participants were patients attending an HIV-specialized clinic located within the County’s referral hospital. To be included in the larger study, individuals had to be adults, 18 to 60 years old, of confirmed seropositive status, attending the HIV-specialized clinic for cART management and monitoring and providing consent for participation. Eligible individuals were excluded from the study if they had acute medical illness or cognitive difficulties at the time of enrolment or administration of questionnaires. Those who could not comprehend and/or communicate in Kiswahili (the national language) which was used during administration of all study instruments, were excluded from participation. The study did not include elderly individuals over 60 years living with HIV because of the increased likelihood of illnesses associated with advancing age, that may have an impact on the quality of life [25].

### Study instruments

For all participants, study instruments were administered via android tablets by interviewers, in the same order and under the same administration environment. Administering research assistants received a 4-day training in research ethics and good interviewing techniques (with role plays) and were familiarized with the tablet-based questionnaires.

#### Sociodemographic and asset index forms

These forms were used to collect information on participant’s age, sex, marital status, level of education, employment, and whom they currently lived with. Socioeconomic status (SES) of study participants was evaluated using an asset index previously used in this setting [26]. A total asset score is calculated, higher scores are indicative of better SES.

#### Health history and treatment characteristics

A health history form was used to capture information about patient’s self-reported history of current: smoking, khat or alcohol use, and presence of any opportunistic infection or chronic illness (that they were made aware of by their physician). Also, patients were asked about disclosure of their HIV status, their satisfaction with current state of care and perception about clinic accessibility using a “Yes” or “No” response option. In responding to the latter item, participants were asked to consider the distance between where they currently lived and the location of the HIV-clinic.

#### Clinical characteristics

A clinical record form was used to extract information on cART regimen, viral load (within the last 1 year), CD4 cell count, HIV clinical stage, dates of HIV diagnosis and cART initiation, most recent weight and height (for body mass index calculation). All participating patients gave prior consent for the above information to be extracted from the clinic’s medical records. We point out here that there were major missing participant data on viral load from the database (*n* = 145) with no follow-up record of CD4 cell count for all participants.

The 9-item Patient Health Questionnaire (PHQ-9) [27] was used as a measure of depressive symptoms. It is rated on a Likert scale of “0” (not at all) to “3” (nearly every day). Scores of these 9 items are summed to derive a total score which ranges from 0 to 27. Consistent with previous studies from SSA [28–30], a cut-off score of ≥10 was used in the present study to define a positive screen for depressive symptoms. PHQ-9 has previously been used with PLWHA in Kenya showing acceptable internal consistency [31].

The 12-item HIV stigma scale [32] was used as a measure of perceived HIV-related stigma. This scale assesses stigma under four dimensions: i) personalised stigma; ii) disclosure concerns; iii) negative self-image; and iv) concerns with public attitudes. Items are rated on a 4-point Likert scale from “1” (strongly disagree) to “4” (strongly agree). A total score is derived from summation of item scores and ranges between 12 and 48. Higher scores indicate a greater level of perceived HIV-related stigma. In its original validation, this scale had acceptable reliability [32].

The Jefferson Scale of Patient’s Perceptions of Physician Empathy (JPPPE) [33] was used to assess patients’ perceptions of their physician’s empathy. The 5 items are rated on a response set like the HIV stigma scale above but with an additional option of being “neutral”. The maximum total score is 25. The higher the score, the greater the perception of physician empathy. There is reported evidence in support of the validity and reliability of this scale [34].

Morisky, Green and Levine Medication Adherence Questionnaire [35]: This 4-item self-report scale was used to assess adherence to cART using “yes” (1) or “no” (0) response options. Responses of “no” to all the 4 items (total score of 0) indicates high level of medication taking behaviour. A score of 1 or 2 to all the 4 items indicates medium adherence whereas a score ≥ 3 is indicative of poor/low adherence to medication.

Functional assessment of HIV infection (FAHI) questionnaire [17] is an HIV-specific measure that assesses HRQoL using 5 sub-scales of: Physical wellbeing, Emotional wellbeing, Functional and Global wellbeing, Social wellbeing and Cognitive functioning. Sub-scales scores are summed to generate an overall HRQoL score. We have previously adapted and explored the psychometric characteristics of a Swahili version of this measure [18]. In this study, the Likert response option was modified from 3-point to 5-point as a standard format of administering the FAHI regardless of language version as stipulated by the test-developers. Graphical presentation of response options was used to help participants easily respond to the 5-point Likert options of “0” (not at all) through “4” (very much). With this Likert rating, the possible maximum score after rescaling negative items is 176. Higher scores indicate better HRQoL.

### Instrument translation and cross-cultural adaptation

Following World Health Organization’s guidelines for translation of tools in health research (http://www.who.int/substance_abuse/research_tools/translation/en/), the English versions of questionnaires not previously translated and/or adapted into Kiswahili (HIV-stigma scale, Jefferson’s scale of physician empathy, and the medication adherence questionnaire) were forward translated by two independent bilingual translators to Kiswahili. Back translations into English were then undertaken by two other independent back translators (unaware of the original version). A group of HIV researchers (natives of Kenya, knowledgeable about the Kenyan culture, bilingual and fluent in both English and Kiswahili) and the translators then held a harmonization meeting to review the content, conceptual, semantic and idiomatic equivalence of the questionnaires [36, 37]. The aim was to ensure cultural relevance of the questionnaires to the targeted sample. The final versions were obtained following incorporation of changes resulting from pretesting procedures. The changes were mainly related to administration procedures as follows:

- For the medication adherence scale, it was observed that some participants were hesitant to talk about their medication adherence. Since data collection was conducted within the clinic setting, we presume they feared the clinic personnel would be informed of their non-adherence if disclosed. In the final version of this scale, the following introductory statement was added re-emphasizing confidentiality as explained in the consent information sheet: ‘*The following 4 questions will ask about your adherence to antiretroviral medication. Be assured that the information you give will be treated with utmost confidentiality and will be used for this research only. Your information will not be shared with anyone else here at the clinic, so feel free to talk*’.
- For JPPPE and HIV stigma scale, it was observed that some participants could not comprehend all the response options read out to them. In administering the final versions, all research assistants were instructed to break down the response options into simpler options such as ‘*do you agree or not agree (or additionally for JPPPE ‘are neutral’) with this question*’ and depending with a participant’s response, further probe would follow e.g. ‘*do you strongly agree or simply agree*’.

### Sample size and study power estimation

In the larger cross-sectional study, the sample size of *N* = 450 was calculated based on prevalence estimate of mental health outcome [38]. In the present study, this sample size is > 95% powered (at 5% level of significance) to detect group differences in overall HRQoL mean scores using previously reported means and standard deviation across significant correlates like ethnicity, marital status, employment, CD4 cell count and depressive symptoms, reported in a past study using the FAHI scale [39].

### Study procedures

In the HIV-specialized clinic, usually, health talks (about half-an hour long) covering topics such as nutrition, adherence or disclosure are presented to all patients before the start of a day’s clinic services. One of the research assistants briefly introduced the study at the end of the daily clinic health talks. Patients who showed interest of participation were invited for further detailed explanation about the study and for consenting as they awaited to be seen by the physician. Those who were able to read were given the study information sheet (in Kiswahili) to read on their own. If a consenting patient met inclusion criteria, administration of study instruments was done after they were seen by their physician, or as they awaited to be seen by the physician if the waiting time was long. Questionnaire administration was conducted in private rooms within the clinic setting and the entire interview session lasted between 30 to 45 minutes.

### Statistical analyses

Frequencies, percentages, means and standard deviations were used to describe sample characteristics. In identifying correlates of HRQoL, prognostic modelling was applied [40, 41]. Using this approach, all independent variables which were associated with each of the five sub-scale and overall scores of the FAHI questionnaire at *p* < 0.20, in univariate linear regression analyses, were fitted in a multivariable linear regression model. In the multivariable model building process, all variables with *p* > 0.05 were removed (one at a time) in a backward stepwise selection process. For the final model, the assumptions of least-squared regression model were checked by visual inspection of: scatter plots and graph matrices (linearity), plots of residual versus predicted values (homoscedasticity) and the normal probability plots (normality). Multicollinearity was checked using variance inflation factor. R (version 3.4.1) statistical software package was used to examine test-retest reliability and fit indices (for exploring construct validity) of the slightly modified FAHI. All other analyses were conducted using STATA (version 15.0).

## Results

### Sample characteristics

In total, 512 adult patients attending an HIV-specialized clinic at Kilifi County Hospital for comprehensive care (including cART management) were approached to participate in this study. Forty-four patients declined to participate. Eighteen patients were excluded because they were either over 60 years (*n* = 11), or unable to comprehend and/or communicate in Kiswahili (*n* = 7). Consequently, 450 patients, hereafter referred to as study participants, were consecutively recruited. Table 1 presents the sociodemographic, health, clinical and treatment characteristics of study participants. The mean age of the study participants was 42.7 years (SD = 9.7). Most of the participants had primary level of education (53.1%), were females (79.1%), unemployed (59.8%), living with family (82.4%), in clinical stage 1 of HIV (93.7%), and self-reported high adherence to cART (92.7%).

**Table 1:** Participant characteristics, *n* = 450

Sociodemographic	Mean/N SD/%	
Age – years, mean, SD	42.7	9.7
Sex – Female	356	79.1%
Marital status		
*Never married*	57	12.7%
*Married/cohabiting*	196	43.6%
*Separated/Divorced/Widowed*	197	43.8%
Education		
*None*	123	27.3%
*Primary*	239	53.1%
*Secondary*	66	14.7%
*Tertiary*	22	4.9%
Employment		
*Unemployed/student*	269	59.8%
*Formally employed*	53	11.8%
*Self-employed*	117	26.0%
*Other*	11	2.4%
Currently living with		
*Alone*	69	15.3%
*Family*	371	82.4%
*Relative/friend*	10	2.2%
Asset index score ^a^ – mean, SD	1.2	1.4
**Health characteristics**		
Currently smoking – Yes, missing = 1	44	9.8%
Current use of khat – Yes	20	4.4%
Current alcohol use – Yes, missing = 1	51	11.4%
Any current chronic illness? -Yes	37	8.2%
**Clinical characteristics**		
BMI – kg/m^2^, mean SD, missing = 4	22.4	4.8
2nd line cART regimen, missing = 4	21	4.7%
Viral load > 1000copies/mL, missing = 145	40	13.1%
WHO clinical stage, missing = 5		
*Stage 1*	417	93.7%
*Stage 2*	22	4.9%
*Stage 3*	3	0.7%
*Stage 4*	3	0.7%
Months since HIV diagnosis – mean, SD	96.8	50.2
Months since cART initiation – mean, SD	81.8	47.1
**Treatment characteristics**		
HIV status disclosure – No	27	6.0%
Any current opportunistic infection? - Yes	83	18.4%
Self-reported cART adherence		
High	417	92.7%
Medium/Low	33	7.3%
Clinic accessibility – No, missing = 2	62	13.8%
Satisfaction with current care – No, missing = 1	19	4.2%

Values are n and % unless otherwise stated

*SD* standard deviation, a - score range = 0 to 7, *cART* combination Antiretroviral therapy, *BMI* body mass index

### Participants’ scores on study instruments

Table 2 presents a summary of participants’ scores on the FAHI, PHQ-9, JPPPE, and HIV-stigma scales disaggregated by sex. Among all the study participants, the mean overall FAHI score was 142.98 (SD = 24.56). The median PHQ-9 score was 3 (IQR = 1–7) and using a cut-off score of ≥10, 13.8% (*n* = 62) of participants had depressive symptoms. The mean scores on the JPPPE and HIV stigma scale were 22.4 (SD = 3.3) and 28.4 (SD = 7.7), respectively.

**Table 2:** Participant scores on continuous measures

Measure	Score range	Total sample *N* = 450	Male *n* = 94	Female *n* = 356
FAHI scale				
Physical wellbeing sub-scale	0–40	30.86 (9.19)	33.78 (8.03)	30.09 (9.33)
Emotional wellbeing sub-scale	0–40	31.98 (8.42)	33.03 (7.39)	31.71 (8.65)
Functional Global wellbeing sub-scale	0–52	44.96 (7.05)	45.89 (5.46)	44.72 (7.04)
Social wellbeing sub-scale	0–32	25.70 (6.25)	27.18 (4.88)	25.31 (6.51)
Cognitive function sub-scale	0–12	9.48 (2.84)	9.87 (2.67)	9.38 (2.88)
Overall scale	0–176	142.98 (24.56)	149.76 (19.75)	141.20 (25.41)
PHQ-9 - median (IQR)	0–27	3 (1–7)	2 (0–6)	3 (1–7)
JPPPE	5–25	22.4 (3.3)	22.3 (3.4)	22.5 (3.3)
12-item HIV-stigma scale	12–48	28.4 (7.7)	26.6 (7.4)	28.9 (7.7)

All values are means (SD) unless otherwise stated

*FAHI* functional assessment of HIV infection scale, *PHQ-9* 9 item patient health questionnaire, *IQR* inter quartile range, *JPPPE* Jefferson Scale of Patient’s Perceptions of Physician Empathy

### Psychometric properties of the study instruments

In the present study, the internal consistency of the PHQ-9 as indicated by Cronbach’s alpha was 0.81 (95% CI: 0.78, 0.84) with a test-retest reliability of 0.78 (95% CI: 0.63, 0.87), based on intra-class correlation coefficient (ICC). The internal consistency alphas of the JSPPE and the HIV stigma scale were 0.75 (95% CI: 0.70, 0.81) and 0.81 (95% CI: 0.78, 0.83), respectively. For the modified FAHI, internal consistency alpha, test-retest reliability, convergent and construct validities were all acceptable. Detailed psychometric results of this modified scale are presented as supplementary information. In summary, internal consistency for the overall FAHI scale was high (Cronbach’s alpha = 0.91; 95% CI: 0.89, 0.92) with sub-scale alphas ranging between 0.57 and 0.88. Test-retest reliability was acceptable for the overall FAHI scale (ICC = 0.73) and ranged between 0.48 and 0.80 for the sub-scales. Significant correlations were observed between FAHI scores (sub-scale and overall) with both PHQ-9 and HIV-stigma scale scores (Supplementary information, Table 1) suggesting good convergent validity. Fit indices for investigating construct validity of the FAHI were all within recommended values (see Supplementary information, Figure 1).

### Correlates of HRQoL among adults receiving cART, coastal Kenya

Tables 3 and 4 present the results following univariate and multivariable linear regression analyses. The variable on viral load was not included in the multivariable regression analysis because many observations had missing values (*n* = 145). For all study participants, there were no any current records of CD4 cell count. Few participants had some baseline CD4 data (when they were being enrolled to the clinic). Therefore, regression analyses also missed CD4 cell count information. From the visual inspection of plots, there was evidence to suggest linearity, homoscedasticity, and normality of generated residuals in all the final linear regression models. Variance inflation factors did not suggest multicollinearity problems.

**Table 3:** Univariate linear regression analysis of correlates of HRQoL among adults receiving cART in Coastal Kenya

Independent variables		β-coefficient (95% CI) of HRQoL domains and overall scale as dependent variables					
	N	Physical wellbeing domain	Emotional wellbeing domain	Functional Global wellbeing domain	Social wellbeing domain	Cognitive function domain	Overall HRQoL
Age	450	−0.03 (− 0.12, 0.06)	0.11** (0.03, 0.19)	0.10*** (0.03, 0.17)	0.07** (0.01, 0.13)	0.01 (−0.02, 0.03)	0.27** (0.03, 0.50)
Sex	450						
Female		Ref	Ref	Ref	Ref	Ref	Ref
Male		3.69*** (1.62, 5.77)	1.33* (−0.59,3.24)	1.17* (− 0.43, 2.78)	1.87** (0.46, 3.29)	0.49* (− 0.15, 1.14)	8.56*** (3.01, 14.10)
Marital status	450						
Married/cohabiting		Ref	Ref	Ref	Ref	Ref	Ref
Separated/divorced/widowed		−.165* (−3.47, 0.17)	−2.32** (− 3.98, − 0.67)	−1.87** (− 3.25, − 0.48)	−1.70** (− 2.93, − 0.48)	− 0.25 (− 0.81, 0.32)	−7.79*** (− 12.60, − 2.98)
Never married		0.18 (− 2.53, 2.90)	− 3.07** (− 5.54, − 0.61)	−2.84** (− 4.91, − 0.77)	− 3.06** (− 4.88, − 1.24)	− 0.66* (− 1.50, 0.18)	−9.45** (− 16.63, − 2.27)
Education	450						
Tertiary		Ref	Ref	Ref	Ref	Ref	Ref
Secondary		−2.88 (− 7.25, 1.50)	− 2.02 (−6.09, 2.06)	0.68 (− 2.74, 4.10)	− 0.11 (− 3.13, 2.92)	0.26 (− 1.12, 1.63)	− 4.06 (− 15.91, 7.79)
Primary		−4.17** (−8.13, − 0.21)	− 1.36 (− 5.04, 2.33)	1.09 (− 2.00, 4.19)	0.65 (− 2.09, 3.39)	0.49 (− 0.75, 1.74)	− 3.30** (− 14.02, 7.42)
None		−7.00** (− 11.12, − 2.89)	−2.66* (− 6.49, 1.17)	0.38 (− 2.83, 3.59)	0.02 (− 2.83, 2.87)	0.01 (− 1.29, 1.29)	− 9.26* (− 20.40, 1.88)
Employment	450						
Formally employed		Ref	Ref	Ref	Ref	Ref	Ref
Self-employed		−1.25 (− 4.22, 1.72)	− 0.70 (− 3.45, 2.04)	− 0.04 (− 2.33, 2.26)	0.32 (− 1.70, 2.35)	0.26 (− 0.67, 1.19)	−1.41 (− 9.38, 6.56)
Other forms of employment		− 0.10 (− 6.04, 5.84)	1.42 (− 4.07, 6.92)	0.52 (− 4.07, 5.11)	4.96** (0.91, 9.01)	0.95 (− 0.90, 2.80)	7.75 (− 8.20, 23.71)
Unemployed		−3.35** (− 6.05, − 0.66)	− 0.44 (− 2.94, 2.05)	−1.33 (− 3.41, 0.75)	0.01 (− 1.83, 1.84)	− 0.04 (− 0.88, 0.80)	− 5.16* (− 12.39, 2.08)
Currently living with	450						
Family		Ref	Ref	Ref	Ref	Ref	Ref
Relative/friend		−1.22 (−7.02, 4.58)	−.191 (− 5.48, 5.10)	0.80 (−3.65, 5.24)	− 1.79 (− 5.71, 2.13)	− 0.90 (− 2.69, 0.88)	−3.31 (− 18.77, 12.15)
Alone		0.43 (− 1.94, 2.80)	−1.99* (− 4.15, 0.18)	−1.02 (− 2.83, 0.80)	−1.66** (− 3.26, − 0.05)	− 0.66** (− 1.39, 0.07)	− 4.90* (− 11.22, 1.43)
Socioeconomic asset index	450	0.79** (0.17, 1.41)	0.10 (− 0.47, 0.67)	0.46* (− 0.02, 0.94)	0.55** (0.13, 0.98)	0.07 (− 0.13, 0.26)	1.96** (0.31, 3.62)
Currently smoking	449						
No		Ref	Ref	Ref	Ref	Ref	Ref
Yes		−1.34 (− 4.20, 1.53)	− 0.67 (−3.30, 1.96)	− 0.48 (− 2.68, 1.73)	− 0.23 (− 2.18, 1.73)	− 0.27 (− 1.15, 0.61)	−2.98 (− 10.65, 4.69)
Current khat use	450						
No		Ref	Ref	Ref	Ref	Ref	Ref
Yes		3.55* (−0.57, 7.67)	3.16* (− 0.62, 6.93)	1.61 (−1.56, 4.78)	0.47 (−2.34, 3.28)	− 0.03 (− 1.31, 1.24)	8.75 (− 2.27, 19.78)
Current alcohol use	449						
No		Ref	Ref	Ref	Ref	Ref	Ref
Yes		1.59 (−1.10, 4.27)	−0.11 (− 2.58, 2.36)	0.54 (− 1.52, 2.61)	− 0.40 (− 2.23, 1.43)	0.05 (− 0.78, 0.88)	1.68 (− 5.52, 8.87)
Disclosure	450						
Yes		Ref	Ref	Ref	Ref	Ref	Ref
No		−2.61* (−6.19, 0.97)	−6.44*** (−9.67, −3.21)	−4.06*** (− 6.79, −1.33)	−5.04*** (− 7.43, − 2.64)	−0.36 (− 1.46, 0.75)	−18.50*** (− 27.94, − 9.06)
Clinic accessible	448						
Yes		Ref	Ref	Ref	Ref	Ref	Ref
No		−3.39** (−5.85, −0.94)	− 3.05** (− 5.30, − 0.80)	−3.09*** (−4.96, −1.21)	− 2.20** (− 3.86, − 0.53)	−1.43*** (− 2.18, − 0.67)	−13.15*** (− 19.66, − 6.64)
Satisfaction with current care	449						
Satisfied		Ref	Ref	Ref	Ref	Ref	Ref
Not satisfied		−1.48 (− 5.71, 2.76)	−0.91 (−4.80, 2.97)	− 4.21** (−7.44, − 0.98)	−4.19*** (− 7.05, − 1.33)	− 0.17 (− 1.49, 1.14)	−10.97* (− 22.27, 0.32)
Any current opportunistic infection	450						
No		Ref	Ref	Ref	Ref	Ref	Ref
Yes		−3.30*** (−5.47, −1.12)	−4.01*** (− 5.99, −2.03)	− 1.37* (− 3.06, 0.31)	−0.25 (− 1.74, 1.24)	− 0.27 (− 0.95, 0.41)	−9.20*** (− 15.01, − 3.39)
Any current chronic illness	450						
No		Ref	Ref	Ref	Ref	Ref	Ref
Yes		−2.45* (−5.53, 0.66)	−0.77 (− 3.62, 2.06)	−0.46 (− 2.84, 1.92)	−1.23 (− 3.34, 0.87)	− 0.26 (− 1.22, 0.70)	−5.17 (− 13.45, 3.11)
Depressive symptom	450						
No		Ref	Ref	Ref	Ref	Ref	Ref
Yes		− 12.85*** (− 15.02, − 10.69)	−12.21*** (− 14.17, − 10.25)	−6.82*** (− 8.61, − 5.03)	− 2.66*** (−4.32, − 1.00)	− 3.20*** (− 3.90, − 2.49)	−37.75*** (− 43.35, − 32.15)
HIV stigma	450	− 0.37*** (− 0.47, − 0.26)	− 0.54*** (− 0.62, − 0.45)	−0.20*** (− 0.28, − 0.11)	−0.22*** (− 0.29, − 0.15)	−0.11*** (− 0.14, − 0.07)	−1.42*** (− 1.69, − 1.16)
Duration since HIV diagnosis	450	− 0.01 (− 0.03, 0.01)	0.01* (− 0.01, 0.03)	−0.01 (− 0.02, 0.01)	−0.01 (− 0.01, 0.01)	−0.01 (− 0.01, 0.01)	− 0.01 (− 0.05, 0.04)
Duration on cART	450	0.01 (−.01, 0.02)	0.02** (0.01, 0.04)	−0.01 (− 0.02, 0.01)	0.01 (− 0.01, 0.01)	−0.01 (− 0.01, 0.01)	0.02 (− 0.02, 0.07)
cART regimen	446						
First line		Ref	Ref	Ref	Ref	Ref	Ref
Second line		−1.14 (− 5.19, 2.90)	− 4.24** (− 7.93, − 0.55)	1.01 (− 2.08, 4.11)	0.98 (− 1.78, 3.73)	− 0.72 (− 1.97, 0.53)	−4.11 (− 14.93, 6.71)
ART adherence	450						
High		Ref	Ref	Ref	Ref	Ref	Ref
Medium/ Low		−4.92*** (− 8.15, −1.68)	− 5.02*** (−7.97, − 2.06)	−1.66* (− 4.17, 0.84)	0.85 (− 1.37, 3.07)	−1.40*** (− 2.41, −0.40)	−12.15*** (− 20.81, − 3.48)
Viral Load	305						
≤1000 copies		Ref	Ref	Ref	Ref	Ref	Ref
> 1000 copies		−0.15 (− 3.12, 2.83)	−1.65 (−4.39, 1.09)	− 2.18* (− 4.57, 0.21)	0.48 (− 1.66, 2.63)	−0.74* (− 1.71, 0.23)	−4.24 (− 12.29, 3.81)
WHO staging	445						
Stage 1		Ref	Ref	Ref	Ref	Ref	Ref
Stage 2		−0.83 (−4.80, 3.13)	−1.24 (− 4.87, 2.39)	−2.37* (− 5.40, 0.65)	0.90 (− 1.80, 3.60)	0.62 (− 0.60, 1.84)	− 2.92 (− 13.51, 7.67)
Stage 3		4.17 (− 6.38, 14.67)	5.08 (− 4.54, 14.70)	3.61 (− 4.40, 11.62)	4.40 (− 2.74, 11.55)	0.87 (− 2.37, 4.10)	18.13 (−9.92, 46.17)
Stage 4		2.17 (−8.34, 12.67)	5.41 (− 4.20, 15.03)	1.28 (− 6.73, 9.29)	0.07 (−7.08, 7.22)	− 2.13 (− 5.37, 1.10)	6.79 (− 21.25, 34.84)
Physician empathy	450	0.37** (0.11, 0.62)	0.31** (0.07, 0.54)	0.73*** (0.54, 0.91)	0.68*** (0.52, 0.84)	0.15*** (0.07, 0.23)	2.23*** (1.58, 2.89)
BMI	446	0.29*** (0.11, 0.46)	0.20** (0.04, 0.36)	0.23*** (0.10, 0.37)	0.04 (−0.08, 0.17)	0.04 (−0.02, 0.09)	0.80*** (0.33, 1.27)

*HRQoL* health-related quality of life, *cART* combination antiretroviral therapy, *BMI* body mass index

*p < 0.20

**p < 0.05

***p < 0.01

**Table 4:** Multivariable linear regression analysis of correlates of HRQoL among adults receiving cART in Coastal Kenya

Independent variables	β-coefficient (95% CI) of HRQoL domains and full-scale as dependent variables					
	Physical wellbeing domain	Emotional wellbeing domain	Functional Global wellbeing domain	Social wellbeing	Cognitive function domain	Overall HRQoL
Age		0.08** (0.02, 0.14)				
Sex						
Female	Ref		Ref	Ref		Ref
Male	3.24** (1.45, 5.03)		1.67* (0.25, 3.08)	2.25** (0.96, 3.53)		9.60** (5.46, 13.74)
Currently living with						
Family		Ref		Ref	Ref	Ref
Relative/friend		2.95 (−1.03, 6.93)		−0.98 (−4.46, 2.50)	−0.18 (− 1.77, 1.41)	5.95 (−5.09, 16.99)
Alone		−2.05* (−3.67, −0.43)		−1.95** (−3.39, − 0.52)	−0.83* (− 1.48, − 0.18)	−6.93** (− 11.47, − 2.39)
Disclosure						
Yes		Ref	Ref	Ref		Ref
No		−4.36** (−6.88, − 1.83)	−3.62** (− 6.02, − 1.21)	−3.96** (− 6.15, − 1.77)		−15.55** (− 22.50, −8.60)
Clinic accessible						
Yes				Ref	Ref	Ref
No				−1.88* (−3.39, −0.38)	−0.97** (− 1.66, − 0.28)	−8.23** (− 13.11, − 3.35)
Any current opportunistic infection						
No		Ref				Ref
Yes		−2.76** (−4.27, −1.24)				−4.54* (−8.80, −0.27)
Depressive symptom						
No	Ref	Ref	Ref		Ref	Ref
Yes	−11.09** (−13.25, −8.94)	−9.99** (− 11.73, − 8.24)	−6.22** (−7.89, −4.56)		−2.65** (−3.35, − 1.95)	−30.23** (− 35.16, − 25.30)
HIV stigma	−0.22** (− 0.31, − 0.12)	−0.40** (− 0.48, − 0.32)		−0.16** (− 0.22, − 0.09)	−0.07** (− 0.10, − 0.04)	−0.86** (− 1.08, − 0.64)
Duration on cART		0.01* (0.01, 0.03)				
Physician empathy	0.24* (0.03, 0.46)		0.67** (0.50, 0.85)	0.60** (0.44, 0.76)	0.10** (0.03, 0.17)	1.59** (1.09, 2.09)
BMI	0.26** (0.11, 0.41)		0.23** (0.11, 0.35)			0.66** (0.32, 1.01)
n of the final model	446	450	446	448	448	445
R-squared for the final model	30.9%	45.8%	25.9%	23.8%	23.1%	51.5%

*HRQoL* health-related quality of life, *cART* combination antiretroviral therapy, *BMI* body mass index

*p < 0.05

**p < 0.01

In the univariate linear regression analyses, neither current smoking nor alcohol use was significantly associated with the sub-scale and overall HRQoL scores of the study participants (at *p* < 0.05) or even at an a priori set *p* < 0.20 for consideration in the multivariable linear regression analyses.

In the multivariable linear regression analyses, having depressive symptoms was strongly associated with lower overall (β = 30.23, 95% CI -35.16, − 25.30; *p* < 0.01) and sub-scale HRQoL scores except for the Social wellbeing domain (Table 4). Similarly, high perceived HIV-stigma scores were significantly associated with lower overall (*β = − 0.86, 95% CI -1.08, − 0.64; p < 0.01*) and sub-scale HRQoL scores except for the Functional and Global wellbeing domain (Table 4).

Participants who reported staying alone compared to those who stayed with family were significantly more likely to have lower overall (*β = − 6.93, 95% CI -11.47, − 2.39; p < 0.01*) and sub-scale HRQoL scores except in the domains of Physical wellbeing and Functional and Global wellbeing (Table 4). Participants who had not disclosed their HIV status were significantly more likely to have lower overall (*β = − 15.55, 95% CI -22.50, − 8.60; p < 0.01*), and sub-scale HRQoL scores except in the domains of Physical wellbeing and Cognitive functioning (Table 4). Participants who found the HIV-clinic inaccessible relative to those who found it accessible were significantly more likely to have lower overall HRQoL scores (*β = − 8.23, 95% CI -13.11, − 3.35; p < 0.01*), lower quality of life scores in the Social wellbeing (*β = − 1.88, 95% CI -3.39, − 0.38; p = 0.01*), and Cognitive functioning (*β = − 0.97, 95% CI -1.66, − 0.28; p < 0.01*) domains, but not for the other sub-scales (Table 4). Presence of any current opportunistic infection was also significantly associated with lower overall HRQoL scores (*β = − 4.54, 95% CI -8.80, − 0.27; p = 0.04*), and lower quality of life score only in the Emotional wellbeing domain (*β = − 2.76, 95% CI -4.27, − 1.24; p < 0.01*) for the studied population.

On the other hand, male sex was significantly associated with better overall (*β = 9.60, 95% CI 5.46, 13.74; p < 0.01*) and sub-scale HRQoL scores except for the Emotional wellbeing and Cognitive functioning domains (Table 4). Higher physician empathy was significantly associated with better overall (*β = 1.59, 95% CI 1.09, 2.09; p < 0.01*) and sub-scale HRQoL scores except for the Emotional wellbeing domain (Table 4). Similarly, a unit increase in BMI was significantly associated with better overall HRQoL score (*β = 0.66, 95% CI 0.32, 1.01; p < 0.01*), better quality of life score in the Physical wellbeing (*β = 0.26, 95% CI 0.11, 0.41; p < 0.01*), and Functional and Global wellbeing (*β = 0.23, 95% CI 0.11, 0.35; p < 0.01*) but not Emotional wellbeing, Social wellbeing, and Cognitive functioning domains.

There were no statistically significant association between any of the proxies for SES (i.e. employment, education and asset index) and HRQoL (sub-scale or overall). Similarly, no statistically significant associations were found between age, duration on cART (in months) and overall HRQoL. However, significant associations were found between both age and duration on cART with Emotional wellbeing domain. As shown in Table 4, one-year increase in age was significantly associated with 0.08-point higher quality of life scores in Emotional wellbeing. Also, a one-month increase of cART use was significantly associated with a 0.01-point higher quality of life scores in the Emotional wellbeing domain.

The final linear regression model consisting of variables on sex, whom the participants currently lived with, disclosure, clinic accessibility, opportunistic infection, symptoms of depression, HIV-related stigma, physician empathy and BMI explained 51.5% of the variance in overall HRQoL. Variance explained by the final sub-scale linear regression models are shown in Table 4.

## Discussion

In this cross-sectional study, a slightly modified FAHI scale with acceptable psychometric properties was used to explore correlates of HRQoL among PLWHA from the Kenyan coast. Consistent with previous findings from SSA [19, 22, 26] and elsewhere [8, 11]; this study found that presence of depressive symptoms and HIV-related stigma are associated with lower HRQoL of PLWHA, both in terms of strength of the association and the number of associated HRQOL domains (4/5 sub-scales and overall HRQoL). Because causality cannot be inferred based on the study design, future studies should explore these consistent findings to help discern the direction of the observed relationship (e.g., whether depressive symptoms cause poor HRQoL or vice-versa). This way, potential interventions, as recommended by Mekuria et al. [19] can be tested meaningfully.

In this study, there was an association between higher physician empathy and better HRQoL both overall and across most sub-scales. Since hardly any study has looked at physician empathy as a determinant of HRQoL in the context of HIV/AIDS, it is difficult to compare our findings. Nonetheless, our finding is comparable to findings from a recent study looking into the association between physician empathy and HRQoL in patients with a chronic condition [42]. There is a need for health care providers of people living with HIV to practice a patient-centred care approach. This involves a model of care that is caring, respectful and compassionate (CRC) which may contribute to a higher quality of life [43, 44]. The tenets of CRC care model are: i) provision of care with empathy, ii) effective communication amongst service providers and between service providers and patients, iii) respecting a patient’s decision in care, and iv) personal satisfaction in serving clients as a health professional.

Following a linear association, higher BMI was strongly associated with better HRQoL of adults living with HIV/AIDS from our setting. This finding corroborates findings from another study conducted in a low-resourced setting [45], but contrasts results from a study done in high-resourced settings [46]. George et al. [46] reported lower odds of having better quality of life with higher BMI (*0.92; 95% CI 0.86, 0.97*). We think that on the Kenyan Coast and other low-resourced settings, where levels of inequality and poverty are high, a higher BMI may just be a proxy marker for better living situation. For PLWHA who are required to eat nutritious food regularly (e.g., up to five servings a day is recommended), higher BMI may be indicative of regular food availability hence better HRQoL. In an Ethiopian study involving PLWHA, Mekuria et al. [19] found that sufficient nutritious food was associated with better HRQoL in most of the domains they assessed. Since in the West there is hardly food insecurity, the contrasting finding by George et al. [46] may be because of body size perception where being overweight or obese is generally negatively perceived and has been associated with outcomes like depression [47].

In this study, demographic characteristics of sex and participant current living situation correlated with HRQoL across 3/5 subscales and overall. Male sex compared to female was significantly associated with better Physical wellbeing, Functional and Global wellbeing, Social wellbeing and overall HRQoL. Males having better HRQoL has also been previously reported elsewhere [8, 48]. Better HRQoL scores among males in this study could be due to lesser societal demands hence better social environment compared to females who in highly patriarchal settings like ours, have additional roles e.g. caregiving and daily house chores yet must cope with HIV disease and its treatment. Living alone compared to living with family (immediate or through marriage) was significantly associated with lower Emotional wellbeing, Social wellbeing, Cognitive functioning and overall HRQoL. However, George et al. [46] comparing quality of life of participants living with family versus those living alone did not find any significant association. We think that a close family relationship contributes to less emotional problems, more social contact and better cognitive functioning (all potentially because of better social support). For instance, living with a stable partner, as family, has been associated with better mental health aspect of quality of life [49]. This finding points out the potential significant role of social support in ensuring the wellbeing of people living with chronic illnesses.

Age as a demographic characteristic was associated with only Emotional wellbeing, but not any other HRQoL domain in this study. In contrast, younger age has been associated with better HRQoL [19, 50]. A potential explanation of our finding is that increasing age could facilitate the development of effective coping strategies among the participants which in turn leads to lesser emotional problems related to living with HIV/AIDS.

Among the treatment characteristics, non-disclosure and presence of any opportunistic infection were significantly associated with lower HRQoL among study participants. Previous studies have also reported opportunistic infection [22, 51] as a correlate of HRQoL. In contrast to our finding, Préau et al. [52] reported that disclosing HIV-status was associated with poorer physical and mental health aspects of quality of life. Mainly because of social support, disclosure of seropositive status could be beneficial for the patient’s mental and physical health [53]. PLWHA should therefore be prepared, encouraged and supported by service providers to voluntarily disclose their HIV status to whom they feel comfortable. If a patient is not willing to disclose, services providers need to respect this decision, being aware that patients can be confronted with stigma and discrimination when disclosure is done involuntarily.

This study found that clinic inaccessibility was associated with lower social wellbeing, cognitive functioning and overall HRQoL. Hardly any study has explored this aspect among PLWHA making it difficult to compare this finding. A Vietnamese study [54] found a significant association between access to health care and HRQoL in univariate but not multivariable analysis. When primary HIV clinics are far, patients incur extra costs of accessing care such as transportation. Because of poverty, sometimes they may be forced to borrow from neighbours, friends or other social networks to cater for such costs. If unable to repay, this may impact on their social relations and overall functioning as they are concerned about repayment but also where next to seek help.

A longer duration on cART, another treatment characteristic, was associated with better emotional wellbeing. Similar findings are reported from a Portuguese study [55], a study conducted in South India [48] and Nigeria [56]. In contrast, a study conducted in the central region of Kenya [57] found longer duration on cART to be negatively associated with physical health summary but no significant associations were reported on the mental health summary. For the observed finding, the authors put forward an argument that treatment fatigue may affect adherence with longer duration on cART. In this study, we argue that continued use of cART gives assurance to the patient of better outcomes e.g. absence (or fewer) HIV-related symptoms hence lesser emotional disturbance. This proposition is strengthened by findings from a qualitative study in Uganda on quality of life among PLWHA [58]. In this study, one participant acknowledged the benefits of cART use by saying “they [cART] have really helped me; I used to be unwell with fevers, had no appetite and felt tired all the time. I feel stronger; have no fevers and my skin and body feel and look better”.

### Study strengths and limitations

This study recruited a large number of study participants. An HIV-specific measure of HRQoL with acceptable psychometric properties was used. The use of face-to-face interviews made it possible to assess, among others, the HRQoL of over a quarter of uneducated participants who would not have been assessed otherwise. However, the study is not without limitations which need to be put into context when interpreting the findings. First, the cross-sectional nature of the study design limits any causal inference of the reported study findings. Second, because many participants had missing information on recent viral load (*n* = 145) and none had follow-up data on CD4 counts, these biomarker data could not be included in regression analyses. In the presence of an overwhelmingly large number of patients, resource limitations for financing regular viral load or CD4 tests for monitoring purposes may be a reason as to why these tests are not done regularly. Future studies, especially from low-resource settings, should budget for an objective investigation (i.e. blood sample collection and testing) of these biological factors posited to be correlates of HRQoL [46]. Third and last, our findings may not be generalizable to adults living with HIV/AIDS in remote areas as study participants were recruited from an HIV specialized clinic within the Kilifi County referral hospital which mainly serves the urban and peri-urban population of Kilifi.

## Conclusion

HRQoL of PLWHA receiving cART on the Kenyan Coast is influenced by several factors simultaneously. Depressive symptoms, HIV-related stigma, non-disclosure of HIV status, living alone, clinic inaccessibility, and presence of any current opportunistic infection were correlates of poor HRQoL. Higher physician empathy, male sex, longer duration on cART, higher BMI and age were correlates of better HRQoL.

In improving HRQoL of PLWHA, Mekuria et al. [19] have previously recommended interventions targeting to increase nutritional aspects, reduce depressive symptoms and HIV-related stigma. Additionally, in this context and similar settings, it is important for programme planners to start thinking about possible ways of making HIV-related services more accessible and strengthening the social support structure of PLWHA. For service providers, being empathetic to clients, addressing emerging opportunistic infections and continually encouraging clients to disclose their HIV-status (at least to trusted few) may go a long way in enhancing the wellbeing and treatment outcomes of PLWHA.

## Supplementary information

**Additional file 1.**

## Data Availability

No additional data are available. Anyone interested in accessing the data reported in this article is free to write to the Data Governance Committee of the KEMRI Wellcome Trust Research Programme who will review the application and advise as appropriate, and ensure that uses are compatible with the consent obtained from participants for data collection. Requests can be sent to the coordinator of the Data Governance Committee using the following email: dgc@kemri-wellcome.org.

## Abbreviations

SSA: Sub Saharan Africa
PLWHA: People living with HIV/AIDS
cART: combination antiretroviral therapy
HRQoL: Health-related quality of life
CD4: Cluster of differentiation 4
CGMR: Centre for Geographic Medicine
SERU: Scientific and ethics review unit
SES: Socioeconomic status
BMI: Body mass index
PHQ-9: 9 Item Patient Health Questionnaire
MINI: Mini International Neuropsychiatric Interview
FAHI: Functional assessment of HIV infection
ICC: Intra-class correlation coefficient
CRC: Caring, respectful, and compassionate
