# Supplementary material for: Correlates of health-related quality of life among adults receiving combination antiretroviral therapy in coastal Kenya
Source: Health Qual Life Outcomes. 2020 Jun 5;18:169. doi: 10.1186/s12955-020-01421-0 (PMC7275333; doi:10.1186/s12955-020-01421-0)

**Supplementary information: Psychometric characteristics of the health-related quality of life measure, the FAHI**

**Tool reliability**

The reliability estimates of the modified FAHI scale were all acceptable. Supplementary Table 1 below presents the sub-scale and overall internal consistency and test-retest reliability of the FAHI scale. Sub-scale internal consistency was acceptable ranging from 0.57 (CF sub-scale; 3 items) to 0.88 (PWB sub-scale; 10 items), and 0.91 for the overall scale (44 items). Test-retest reliability was acceptable ranging from 0.48 (physical wellbeing sub-scale) to 0.80 (social wellbeing sub-scale), and 0.73 for the overall scale.

**Supplementary Table 1: Psychometric characteristics of the modified FAHI questionnaire, subscale and overall**

|  | | **Physical wellbeing** | **Emotional wellbeing** | **Functional global wellbeing** | **Social wellbeing** | **Cognitive functioning** | **FAHI Overall** |
| --- | --- | --- | --- | --- | --- | --- | --- |
| Items | | 10 | 10 | 13 | 8 | 3 | 44 |
| Cronbach’s alpha (95% CI) | | 0.88 (0.86, 0.90) | 0.82 (0.79, 0.86) | 0.76 (0.71, 0.80) | 0.74 (0.70, 0.78) | 0.57 (0.49, 0.64) | 0.91 (0.89, 0.92) |
| Test-retest reliability (95% CI) | | 0.48 (0.26, 0.65) | 0.59 (0.40, 0.73) | 0.79 (0.67, 0.87) | 0.80 (0.68, 0.87) | 0.68 (0.52, 0.80) | 0.73 (0.59, 0.83) |
| Convergent validity$ | |  |  |  |  |  |  |
| Correlation with PHQ-9 | -0.64** | -0.61** | -0.45** | -0.29** | -0.49* | -0.71** |  |
| Correlation with HIV-stigma scale | -0.31** | -0.49** | -0.21** | -0.27** | -0.28** | -0.44** |  |
| Notes: $ Pearson’s correlation coefficient  * Correlation is significant at p<0.05  ** Correlation is significant at p<0.01  **HRQoL** – Health-Related Quality of Life, **SD** – Standard deviation, **PHQ-9** – 9-item Patient Health Questionnaire | | | | | | | |

**Tool validity**

Statistically significant correlations (in the expected direction) were observed between sub-scale and overall FAHI scores with both PHQ-9 and HIV-stigma scale scores (Supplementary Table 1) suggesting good convergent validity. In terms of construct validity, all items had an acceptable factor loading (≥0.3) using a five-factor solution in CFA except for two items: “*I worry about spreading my infection*” (emotional wellbeing sub-scale), and “*I am losing hope in the fight against my illness*” (functional global wellbeing sub-scale). Removal of these items minimally improved FAHI overall internal consistency and as such they were retained. These were also found as problematic items and retained in our previous validation work ([Nyongesa et al., 2017](#_ENREF_36)). In second-order CFA, all factor loadings for the five sub-constructs of HRQoL (i.e. physical, emotional, functional global, social and cognitive wellbeing) on the overall/main construct (HRQoL) were good (≥0.6). Supplementary Figure 1 below presents these factor loadings following CFA. As for the goodness of fit indices, all were within the recommended cut-off (Supplementary Figure 1). These patterns of results suggest good construct validity of the FAHI.

**Supplementary Figure 1: Factor loadings for FAHI items and sub-constructs following five-factor solution and second-order CFA**


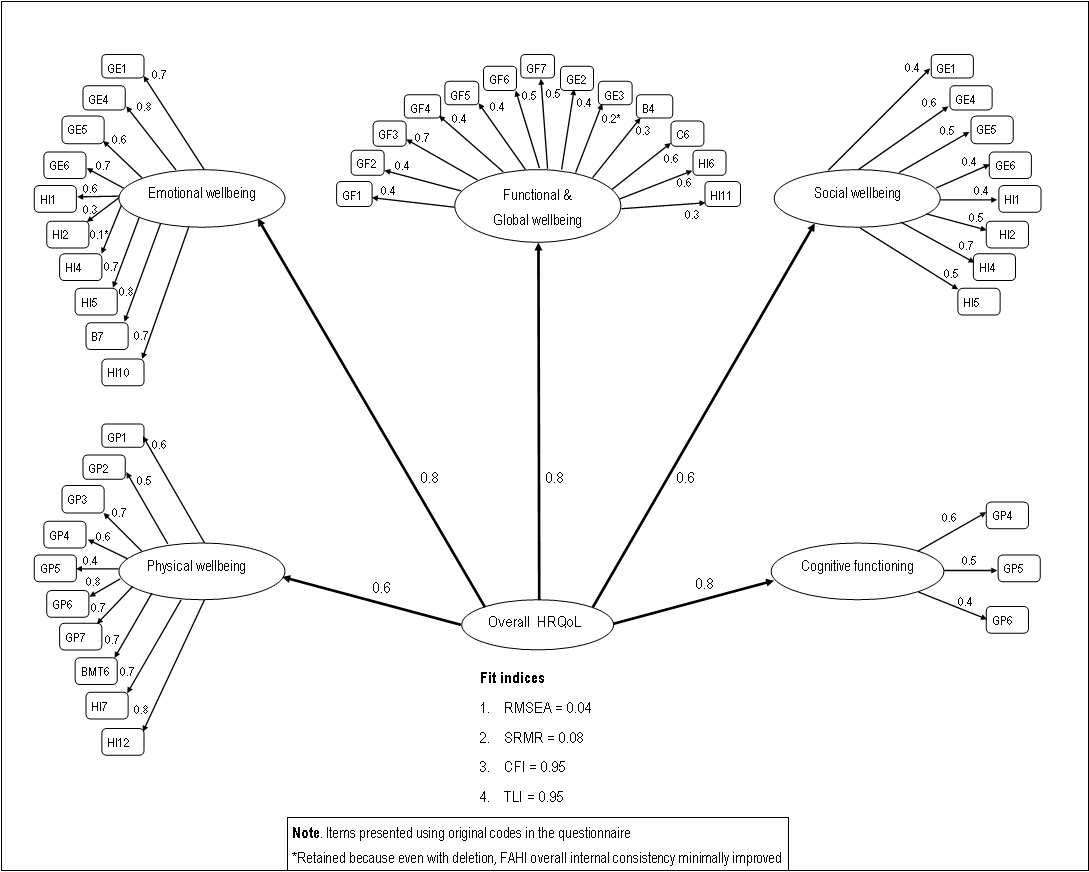

Supplement: Supplementary file 1 — Additional file 1. [file 12955_2020_1421_MOESM1_ESM.docx]
